# Hormone receptor expression profiles differ between primary and recurrent high-grade serous ovarian cancers

**DOI:** 10.18632/oncotarget.15858

**Published:** 2017-03-02

**Authors:** Zheng Feng, Hao Wen, Xingzhu Ju, Rui Bi, Xiaojun Chen, Wentao Yang, Xiaohua Wu

**Affiliations:** ^1^ Department of Gynecological Oncology, Fudan University Shanghai Cancer Center, Shanghai 200032, China; ^2^ Department of Oncology, Shanghai Medical College, Fudan University, Shanghai 200032, China; ^3^ Department of Pathology, Fudan University Shanghai Cancer Center, Shanghai 200032, China

**Keywords:** high-grade serous ovarian cancer, hormone receptors, estrogen receptor, progesterone receptor, androgen receptor

## Abstract

Hormone receptor status assessment is necessary for selecting cancer patients who might potentially benefit from endocrine therapy. To determine whether hormone receptor status changes during tumor progression, we retrospectively examined 107 high-grade serous ovarian cancer (HGSC) patients with paired primary and recurrent tumor specimens. Hormone receptor expression discordance rates between primary and recurrent tumors were as follows: estrogen receptor (ER) 34.9%, progesterone receptor (PR) 12.4%, androgen receptor (AR) 41.7%, follicle stimulating hormone receptor 46.6%, luteinizing hormone receptor 50.5%, and gonadotropin releasing hormone receptor 20.0%. Hormone receptor discordance was not associated with patient survival. The proportion of the PR-ER+AR- subgroup, which exhibited the worst prognosis, was higher in recurrent than primary tumor specimens. Our study demonstrated that paired primary and recurrent HGSC specimens exhibit differing hormone receptor profiles. Thus, to most effectively identify patient-specific therapies, biomarker status re-assessment is required for recurrent patients.

## INTRODUCTION

Ovarian cancer is one of the most common and lethal cancers affecting women globally [1]. After primary debulking or staging surgery, and subsequent platinum-based chemotherapy, most patients relapse within a median of 16 months [2]. Patients with recurrent ovarian cancer are usually offered several consecutive therapies with variable response rates and prognoses [2]. Effective clinicopathological biomarkers to identify the most appropriate alternative therapy for a given patient are urgently needed.

Hormone therapy is considered a salvage therapy for recurrent disease, and endocrine therapy tends to be more efficacious in hormone receptor positive subgroups [3–6]. Hormone receptor status assessment is necessary for selecting patients who would potentially benefit from endocrine therapy. However, hormone receptors or HER2 status can change during breast cancer progression [7–9], although whether hormone receptor expression differs between primary and recurrent ovarian cancers is currently unknown.

High-grade serous ovarian cancer (HGSC) is the main histologic subtype of epithelial ovarian cancers with high hormone receptor levels [10, 11]. Our previous study classified patients into five subgroups with distinctive clinicopathological features via immunohistochemistry [12]. In the present work, we analyzed hormone receptor expression in 107 HGSC patients with paired primary and recurrent tumor specimens, and investigated the clinical significance of hormone receptor status discordance between primary and recurrent HGSC.

## RESULTS

### Patient characteristics

Patient characteristics are provided in Table 1. 107 HGSC patients had a median (range) follow-up time of 42 (4–115) months. 103 (96.3%) patients had advanced stage disease. At the time of primary surgery, 30 (28%) were debulked to no macroscopic residual disease, and 57 (53.3%) were debulked to <1 cm of macroscopic disease. 106 patients received platinum-based adjuvant chemotherapy, and all patients underwent secondary debulking surgery for ovarian cancer recurrence with a median (range) PFS of 15 (5–66) months. At the time of secondary cytoreduction, 60 (56.1%) were debulked to no macroscopic residual disease, and 25 (23.4%) were debulked to <1 cm of macroscopic disease.

**Table 1:** Characteristics of Patients (n=107)

Age at diagnosis, median (range), years		54(36-81)	
Follow-up time, median (range), months		42(4-115)	
Vital status	Died	61	57.0%
	Alive	36	33.6%
	Censored	10	9.3%
FIGO	Early (FIGO I, II)	4	3.7%
	Advanced (FIGO III, IV)	103	96.3%
Primary Cytoreduction	R0	30	28.0%
	0.1-1cm	57	53.3%
	>1cm	20	18.7%
Primary Chemotherapy	Intraperitoneal plus intravenous	39	36.4%
	Intravenous	67	62.6%
	No	1	0.9%
Chemosensitivity	Yes	64	60.4%
	No	41	38.7%
	NA	1	0.9%
Progression-free Survival (range), months		15 (5-66)	
Secondary Cytoreduction	R0	60	56.1%
	0.1-1cm	25	23.4%
	>1cm	22	20.6%
Postoperative chemotherapy	With platinum	90	84.1%
	Without platinum	3	2.8%
	No	14	13.1%
Postoperative chemosensitivity	Yes	23	31.5%
	No	50	68.5%

### Hormone receptor status changes between primary and recurrent specimens

Representative images for hormone receptors are shown in Supplementary Figure 1. Most primary and recurrent ovarian cancers expressed estrogen receptor (ER; 67.0% and 72.9%, respectively) or gonadotropin releasing hormone receptor (GnRHR; 87.9% and 85%, respectively), while progesterone receptor (PR) remained at low levels in both primary and recurrent specimens (9.3% and 6.7%, respectively) (Table 2). Androgen receptor (AR) was downregulated from 33.6% to 17.5%, respectively. Approximately half of the patients expressed follicle stimulating hormone receptor (FSHR) or luteinizing hormone receptor (LHR) in both specimens.

**Table 2:** Expression of hormone receptors by immunohistochemistry (n=107)

Parameters		N (Primary)	%	N (Recurrent)	%
ER	Positive (>10%)	71	67.0%	78	72.9%
	Negative (≤10%)	35	33.0%	29	27.1%
PR	Positive (>10%)	10	9.3%	7	6.7%
	Negative (≤10%)	97	90.7%	98	93.3%
AR	Positive (>10%)	36	33.6%	18	17.5%
	Negative (≤10%)	71	66.4%	85	82.5%
FSHR	Positive (IRS>3)	48	44.9%	57	55.3%
	Negative (IRS<3)	59	55.1%	46	44.7%
LHR	Positive (IRS>3)	43	40.2%	47	45.6%
	Negative (IRS<3)	64	59.8%	56	54.4%
GnRHR	Negative	13	12.1%	15	15.0%
	Weak	17	15.9%	16	16.0%
	Moderate	37	34.6%	33	33.0%
	Strong	40	37.4%	36	36.0%

**Figure 1:**
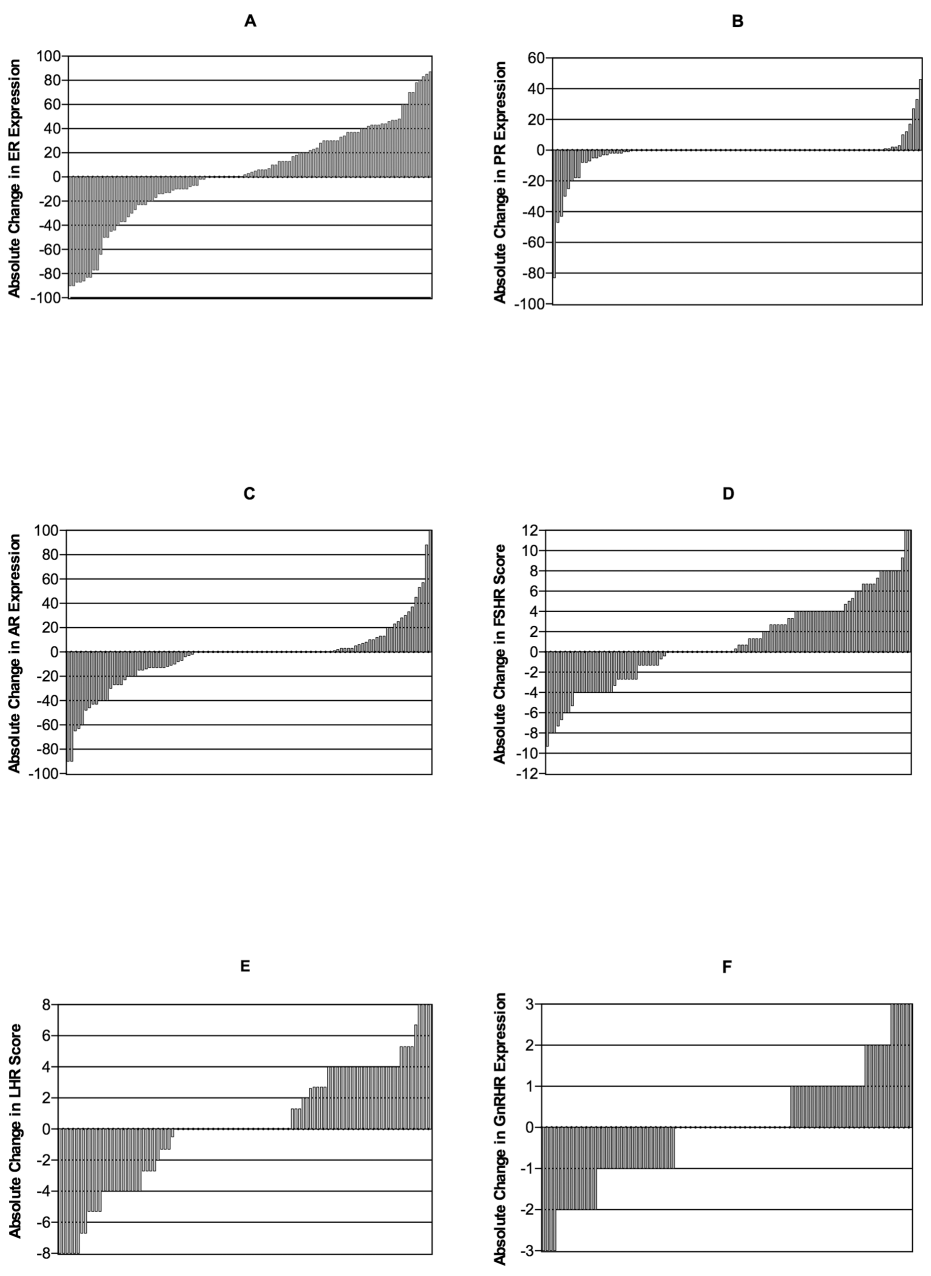
Waterfall plot showing absolute change in ER **(A)**, PR **(B)**, AR **(C)**, FSHR **(D)**, LHR **(E)**, and GnRHR **(F)**. Quantitative hormone receptor change was calculated as quantitative expression in the recurrent specimen minus that in the primary specimen in each case. For ER, PR, AR and GnRHR, this change was the difference in positive staining proportion. For FSHR and LHR, the change was IRS difference. Plots on the horizontal line represent unchanged status. Plots over and under the horizontal line represent increased and decreased positive staining proportion or IRS, respectively.

Hormone receptor discordance rates were as follows: ER 34.9%, PR 12.4%, AR 41.7%, FSHR 46.6%, LHR 50.5%, and GnRHR 20.0% (Table 3). Quantitative hormone receptor change was calculated as quantitative expression in the recurrent specimen minus that in the primary specimen in each case (Figure 1). PR expression remained relatively unchanged, while other hormone receptor levels fluctuated throughout tumor progression.

**Table 3:** Change of hormone receptor expression between paired primary and recurrent specimens

Parameters		N(%)			N(%)		P value
ER	Concordance	69	65.1%	Negative	13	12.3%	0.324
				Positive	56	52.8%	
	Discordance	37	34.9%	Loss	15	14.2%	
				Gain	22	20.8%	
PR	Concordance	92	87.6%	Negative	90	85.7%	0.581
				Positive	2	1.9%	
	Discordance	13	12.4%	Loss	8	7.6%	
				Gain	5	4.8%	
AR	Concordance	60	58.3%	Negative	55	53.4%	0.015
				Positive	5	4.9%	
	Discordance	43	41.7%	Loss	30	29.1%	
				Gain	13	12.6%	
FSHR	Concordance	55	53.4%	Negative	28	27.2%	0.112
				Positive	27	26.2%	
	Discordance	48	46.6%	Loss	18	17.5%	
				Gain	30	29.1%	
LHR	Concordance	51	49.5%	Negative	33	32.0%	0.488
				Positive	18	17.5%	
	Discordance	52	50.5%	Loss	23	22.3%	
				Gain	29	28.2%	
GnRHR	Concordance	80	80.0%	Negative	4	4.0%	0.824
				Positive	76	76.0%	
	Discordance	20	20.0%	Loss	11	11.0%	
				Gain	9	9.0%	

### Prognostic impact of hormone receptor changes

In survival analyses, patients whose recurrent specimens showed hormone receptor upregulation were compared with those whose recurrent specimens showed downregulation. Similarly, patients with hormone receptor expression loss in recurrent specimens were compared with patients who showed hormone receptor upregulation. Hormone receptor discordance was not associated with PFS or OS (Table 4).

**Table 4:** Univariate analyses of hormone receptor discordance with PFS and OS

Parameters		PFS		OS	
		HR	p value	HR	p value
ER discordance	Gain *vs* Negative	0.689(0.316-1.499)	0.347	0.464(0.194-1.108)	0.084
	Loss *vs* Positive	0.933(0.507-1.718)	0.824	0.777(0.340-1.772)	0.548
PR discordance	Gain *vs* Negative	1.598(0.579-4.414)	0.366	0.877(0.212-3.623)	0.856
	Loss *vs* Positive	0.787(0.151-4.109)	0.776	1.776(0.188-16.756)	0.616
AR discordance	Gain *vs* Negative	1.159(0.604-2.225)	0.656	0.570(0.222-1.464)	0.243
	Loss *vs* Positive	0.423(0.153-1.171)	0.098	0.321(0.088-1.177)	0.086
FSHR discordance	Gain *vs* Negative	0.841(0.481-1.472)	0.545	0.715(0.346-1.478)	0.365
	Loss *vs* Positive	1.787(0.931-3.430)	0.081	1.254(0.568-2.772)	0.576
LHR discordance	Gain *vs* Negative	1.196(0.699-2.045)	0.513	1.208(0.631-2.315)	0.568
	Loss *vs* Positive	0.875(0.447-1.715)	0.698	1.813(0.727-4.520)	0.202
GnRHR discordance	Gain *vs* Negative	0.808(0.206-3.170)	0.760	0.639(0.140-2.909)	0.562
	Loss *vs* Positive	1.290(0.638-2.610)	0.479	0.900(0.354-2.287)	0.825

We then analyzed the distribution of hormone receptor-based molecular subgroups according to our previous study (Table 5) [12]. As we previously reported, a trend of increasing risk of death was observed for the following four subgroups: PR-ER-AR+, PR+, PR-ER+AR+, PR-ER-AR- and PR-ER+AR- [12]. In our current study, the proportion of PR-ER+AR- cases increased in recurrent vs. primary specimens (54.9% vs. 37.7%, P=0.020).

**Table 5:** Distribution of hormone receptor based molecular subgroups

Subgroup	Primary		Recurrent		p value
	N	%	N	%	
PR-ER-AR+	5	4.7%	2	2.0%	0.020
PR+	10	9.4%	7	6.9%	
PR-ER+AR+	24	22.6%	14	13.7%	
PR-ER-AR-	27	25.5%	23	22.5%	
PR-ER+AR-	40	37.7%	56	54.9%	

## DISCUSSION

Our study demonstrated hormone receptor status instability in a portion of HGSC patients during ovarian cancer progression. We also associated patient risk of death with tumor hormone receptor status. Receptor status discordance between primary and recurrent breast cancers has been addressed in recent work [19]. Approximately 10–40% of breast cancer patients exhibited unstable receptor statuses during tumor progression [7–9]. Different mechanisms for receptor discordance have been proposed, such as intratumoral heterogeneity and selection through previous treatments [20, 21]. Receptor status instability has also been correlated with patient survival, and could influence therapeutic decision making in the management of recurrent patients [7–9].

The present study identified hormone receptor status discordance between paired primary and recurrent ovarian cancers for the first time. Furthermore, we observed that the proportion of the PR-ER+AR- subgroup, which had the worst prognosis, increased in recurrent vs. primary HGSC cases.

Primary and recurrent ovarian cancer tissue samples collected from the same patient may exhibit distinctive morphological, molecular, and/or genetic features [22, 23]. Marques, *et al*. [23] found that chemotherapy reduced PARP1 expression in ovarian cancer, while Despierre, *et al*. [22] reported that folate receptor alpha expression remained unchanged in epithelial ovarian cancer after chemotherapy. Our results indicate that recurrent ovarian cancers present a more aggressive phenotype compared with primary tumors. Further investigations should focus on mechanisms that drive genetic disparity between primary and recurrent ovarian cancers.

Hormone therapy (tamoxifen, aromatase inhibitors, and others) is considered a salvage therapy for recurrent ovarian cancer patients, although results have been unsatisfactory [3–5]. However, a phase II study of letrozole in ER+ relapsed ovarian cancer patients had more promising response rates [6]. As hormone receptor status may change during ovarian cancer progression, endocrine therapy administration should be based on hormone receptor status in recurrent patients. Additionally, FSHR or GnRHR targeted agents have been developed using corresponding ligands as targeting moieties [24, 25]. Targeted therapy relies on corresponding receptor expression, and biopsies are necessary to confirm this expression.

In conclusion, our study demonstrated that paired primary and recurrent ovarian cancer specimens exhibit discordant hormone receptor statuses. Thus, biomarker status re-assessment in recurrent patients is required to most effectively identify patient-specific therapies.

## MATERIALS AND METHODS

### Clinical data

This study was conducted according to the Declaration of Helsinki and was approved by the Committee at Fudan University Shanghai Cancer Center. Written informed consent was obtained from all study participants. Our retrospectively study included 107 women who underwent both primary staging or debulking surgery and secondary cytoreduction to treat HGSC at Fudan University Shanghai Cancer Center between April 2005 and June 2013. Patients were excluded if they had received neoadjuvant therapy prior to primary surgery, were found to have other histological diagnoses on pathological review, or if paraffin-embedded tissue samples were not available.

Clinical and pathological data were obtained from medical records, cancer registries, and pathology reports. Patient characteristics, including age, FIGO stage, surgical outcomes, date of primary and secondary surgeries, date of progression or recurrence, date of last follow-up, and disease status at last contact, were collected. Patient follow-up for this study ended on December 31, 2014.

R0 was defined as the absence of macroscopic residual disease (RD) after surgery. Chemosensitivity was defined as a time interval of six months or longer between completion of platinum-based chemotherapy and detection of relapse. Chemoresistance was defined as disease progression during adjuvant chemotherapy or within the six-month interval between completion of platinum-based chemotherapy and detection of relapse. Progression-free survival (PFS) was defined as the time interval between primary surgery and disease progression or recurrence. Overall survival (OS) was defined as the time interval between primary surgery and death or last follow-up.

### Tissue microarray and immunohistochemistry

Histological diagnoses were based on the WHO criteria [13], and all paired primary and recurrent specimen slides were reviewed by two experienced gynecologic pathologists. A microarray (1 mm) with triplicate tissue samples from each tumor was prepared [11, 14]. We collected ovarian masses as the primary specimens. We also collected recurrence specimens: 92/107 (86.0%) from pelvic masses, 13/107 (12.1%) from metastatic lymph nodes, and 2 (1.9%) from isolated thoracic masses. Intra-class correlation coefficient (ICC) was calculated to evaluate the internal consistency of immunoscores of three cores from each individual tumor sample. Cronbach's α indexes were approximately 0.9, which meant that there were no differences in parameter expression among the different cores. Sections (3μm) of the completed tissue microarray were analyzed by standard immunohistochemistry methods. Immunohistochemical staining was performed in all cases for ER and PR using a Ventana Benchmark XT autostainer (Ventana Medical Systems Inc., Tucson, AZ, USA). AR, FSHR, LHR and GnRHR staining was performed using the Envision horseradish peroxidase system following the manufacturer's protocol (DAKO EnVision System K5007). Primary antibodies used in this study were as follows: ER (ERα, Roche, Germany, SP1), PR (Roche 1E2), AR (Abcam, UK, ab133273, 1:100), FSH-R (Abcam ab150557, 1:100), LH-R (Santa Cruz, USA, sc-25828, 1:40), and GnRH-R (Abcam ab183079, 1:50). Negative (no primary antibody) and positive (according to the primary antibody instructions) controls were included in each staining run.

Results were blindly and independently judged, evaluated, and scored by two experienced gynecologic pathologists. Results were reported as the numerical means of the triplicate scores. Hormone receptor expression was determined using the following criteria: ER, PR, and AR levels: >10% of cells showing positive nuclear staining of any intensity was considered positive [15, 16]. FSHR and LHR levels: evaluation of the cytoplasmic staining reaction was performed in accordance with the immunoreactive score (IRS). IRS was defined as staining intensity (SI) multiplied by the percentage of positive cells (PP). SI was defined as 0 (negative), 1 (weak), 2 (moderate), and 3 (strong). PP was defined as 0 (negative), 1 (≥10% positive cells), 2 (11–50% positive cells), 3 (51–80% positive cells) and 4 (>80% positive cells). IRS≥3 was considered positive [17]. GnRHR level: Cytoplasmic GnRHR staining was recorded as negative, weak, moderate or strong. Staining of any intensity was considered positive [18].

Discordance rate was defined as the proportion of patients who displayed differential hormone receptor status between primary and recurrent specimens. Discordance included cases in which a primary sample presented as positive for a particular hormone receptor, but recurrent specimens were negative, and cases that initially presented as negative, but turned positive in recurrent specimens.

### Statistical analyses

SPSS software (version 21.0) and GraphPad Prism (version 6.0) were used for statistical analyses. Descriptive statistics were used for demographic data and were summarized as means ± standard deviations (SD), medians with interquartile ranges (IQRs) or ranges, or frequencies with percentages. Categorical data were compared via chi-square or Fisher's exact tests as appropriate. A two-tailed McNemar test was used to evaluate biomarker discordance in the same patient before and after chemotherapeutic treatment. OS was analyzed via the Cox regression method, which is expressed as hazard ratios (HRs). P<0.05 was considered statistically significant, and all reported P-values were 2-sided.

## SUPPLEMENTARY MATERIALS FIGURES


