# Supplementary material for: Hormone receptor expression profiles differ between primary and recurrent high-grade serous ovarian cancers
Source: Oncotarget. 2017 Mar 2;8(20):32848–55. doi: 10.18632/oncotarget.15858 (PMC5464832; doi:10.18632/oncotarget.15858)
Supplement: Supplementary file 1 [file oncotarget-08-32848-s001.pdf]

Hormone receptor expression profiles differ between primary and recurrent high-grade serous ovarian cancers

SUPPLEMENTARY FIGURE

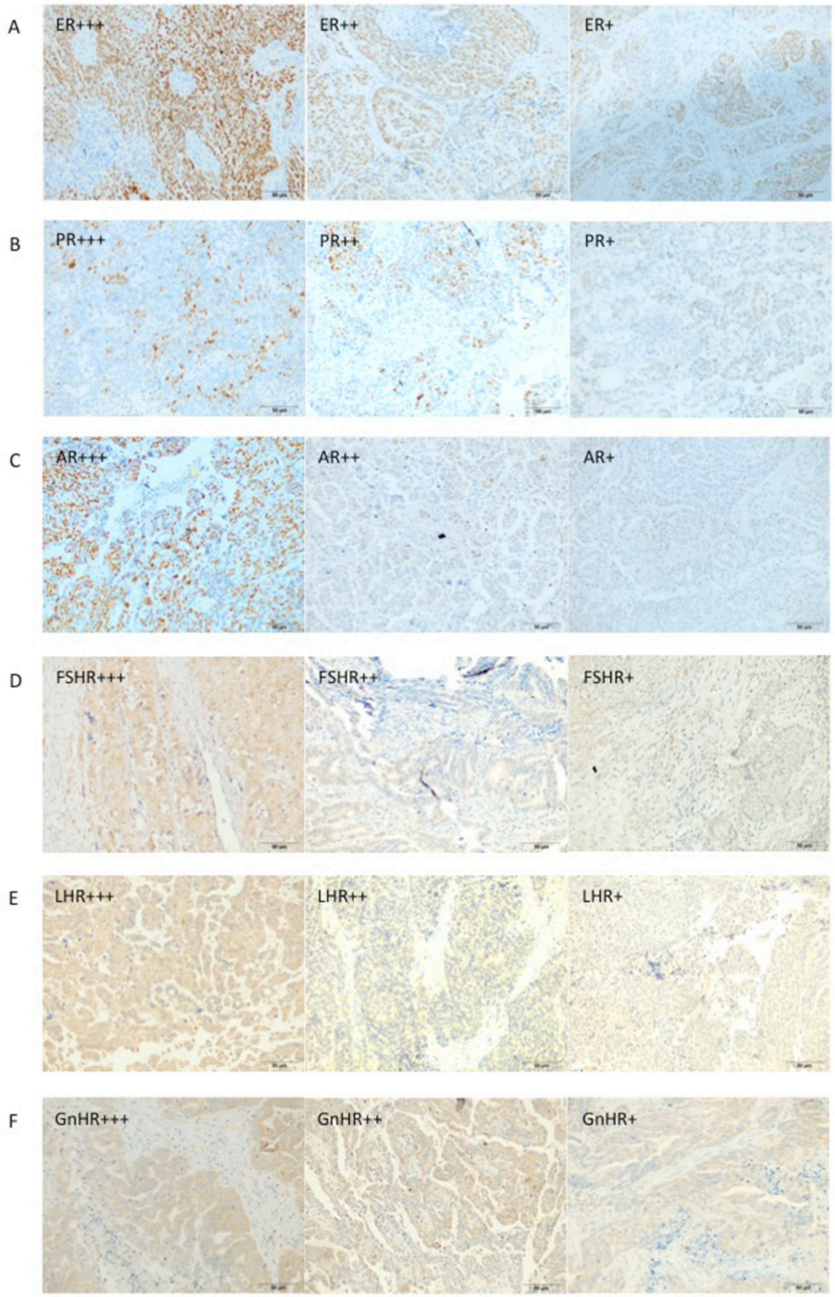

Supplementary Figure 1: Representative positive staining for ER (A), PR (B), AR (C), FSHR (D), LHR (E) and GnRHR (F) at 200x magnification.
